# Modified nusinersen intrathecal injection method: inclusion of a septal needle-free closed infusion connector

**DOI:** 10.3389/fneur.2023.1234442

**Published:** 2023-09-21

**Authors:** Yani Zhang, Kelu Zheng, Cuili Liang, Ruidan Zheng, Jinghui Chen, Minyan Jiang, Zhizi Zhou, Yuan Zhao, Min Rao, Sida Yang, Wenxiong Chen, Li Liu

**Affiliations:** ^1^Department of Neurology, Guangzhou Women and Children’s Medical Center, Guangzhou Medical University, Guangzhou, China; ^2^Department of Endocrinology and Genetic Metabolism, Guangzhou Women and Children’s Medical Center, Guangzhou Medical University, Guangzhou, China; ^3^Department of Anesthesiology and Perioperative Medicine, Guangzhou Women and Children’s Medical Center, Guangzhou Medical University, Guangzhou, China

**Keywords:** modified, intrathecal injection, infusion connector, nusinersen, spinal muscular atrophy

## Abstract

**Objective:**

Nusinersen, an extremely expensive biologic drug (around 100,000 US$ per dose) that needs to be administered intrathecally, is approved for the treatment of 5q-spinal muscular atrophy (SMA). Because of the low muscle tone of the back muscles of pediatric SMA patients, especially type 1 SMA patients, the safe, effective, and fast execution of sheath injection is needed. Therefore, a modified intrathecal injection method was developed accordingly. This paper aims to describe the applicability and safety of this modified method.

**Methods:**

The modified intrathecal injection method (MIIM) mainly includes a septal needle-free closed infusion connector between the lumbar puncture needle and the syringe, besides the procedures of routine lumbar puncture. Its applicability and safety were evaluated through clinical observation.

**Results:**

A total of 92 children with SMA have successfully received nusinersen treatment at our hospital using the modified method since 2019 without obvious adverse events related to the modified injection method. Based on the clinical feedback of operators, the advantages of the modified method include successfully injecting the total dose of nusinersen with constant injection rate and a more stable fixation of the puncture needle, as well as making the operator more relaxed. However, compared with the routine method, the procedure of the modified method has additional steps.

**Conclusion:**

The modified intrathecal injection method is an effective and safe method to inject nusinersen when weighing the pros and cons, and it may also be used for administering intrathecal injections of other expensive medicines or for patients with other strict requirements for intrathecal injection.

## Introduction

Spinal muscular atrophy (SMA) is an autosomal-recessive inherited neuromuscular disorder that causes progressive loss of the motor neurons in the anterior horn cell, leading to muscle atrophy and significant disability. The disease affects 1 in 10,000 live births and represents the leading genetic cause of infant mortality (1).

Recently, three disease-modifying treatments have been approved for use in SMA patients, including pre-messenger RNA splicing of SMN2 nusinersen (2), SMN2 splicing modifier risdiplam (3), and gene replacement therapy by onasemnogene abeparvovec (4). Currently, SMA patients are known to survive longer and have improved outcomes.

Nusinersen was approved by the United States Food and Drug Administration (FDA) for the treatment of 5q-mutated SMA in 2016 (2, 5) and introduced in China in 2019. Nusinersen is an extremely expensive medicine (around 100,000 US$ when initially used in China) which needs to be intrathecally injected. Besides the high price of nusinersen, the risk of an unstable lumbar puncture needle caused by the reduced muscle tone of low back muscles especially in type 1 SMA patients leads to the psychological and physical tension of the operator to some extent. Therefore, clinicians at our hospital have found some room for the improvement of intrathecal injection during routine lumbar puncture and developed a modified intrathecal injection method (MIIM) for nusinersen. This paper aims to share the applicability, efficacy, and safety of this modified method.

## Materials and methods

The MIIM was developed and is only performed at our hospital. Hence, this was a single center study. All included children were confirmed with 5q-mutated SMA by gene analysis. Written informed consent was obtained from the patient/parent/guardian regarding which injection method was to be used. Intrathecal injection was performed by five experienced pediatricians with more than 10 years of experience in lumbar puncture.

**Figure 1:**
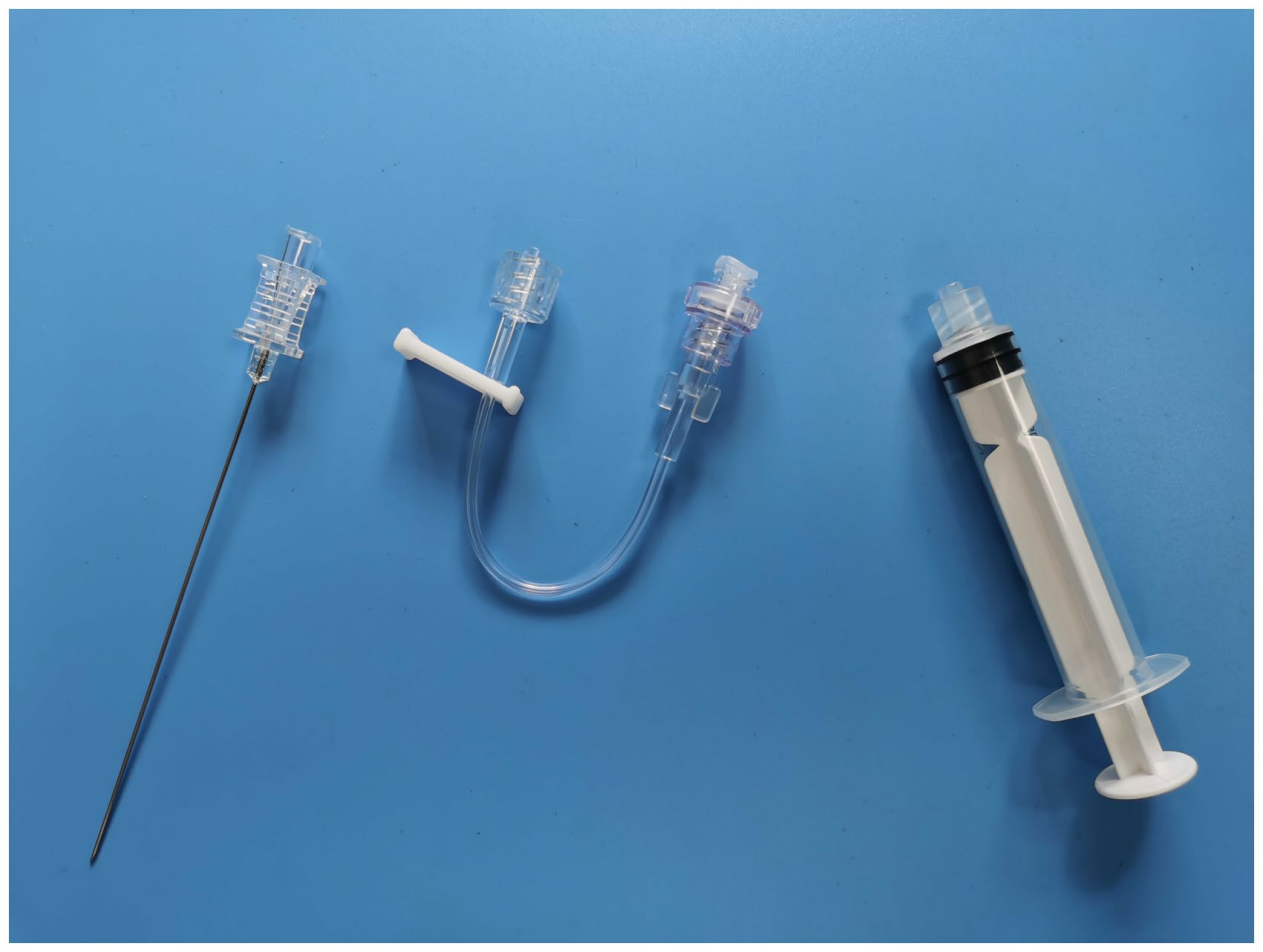
Lumbar puncture needle, septal needle-free closed infusion connector, and syringe.

**Figure 2:**
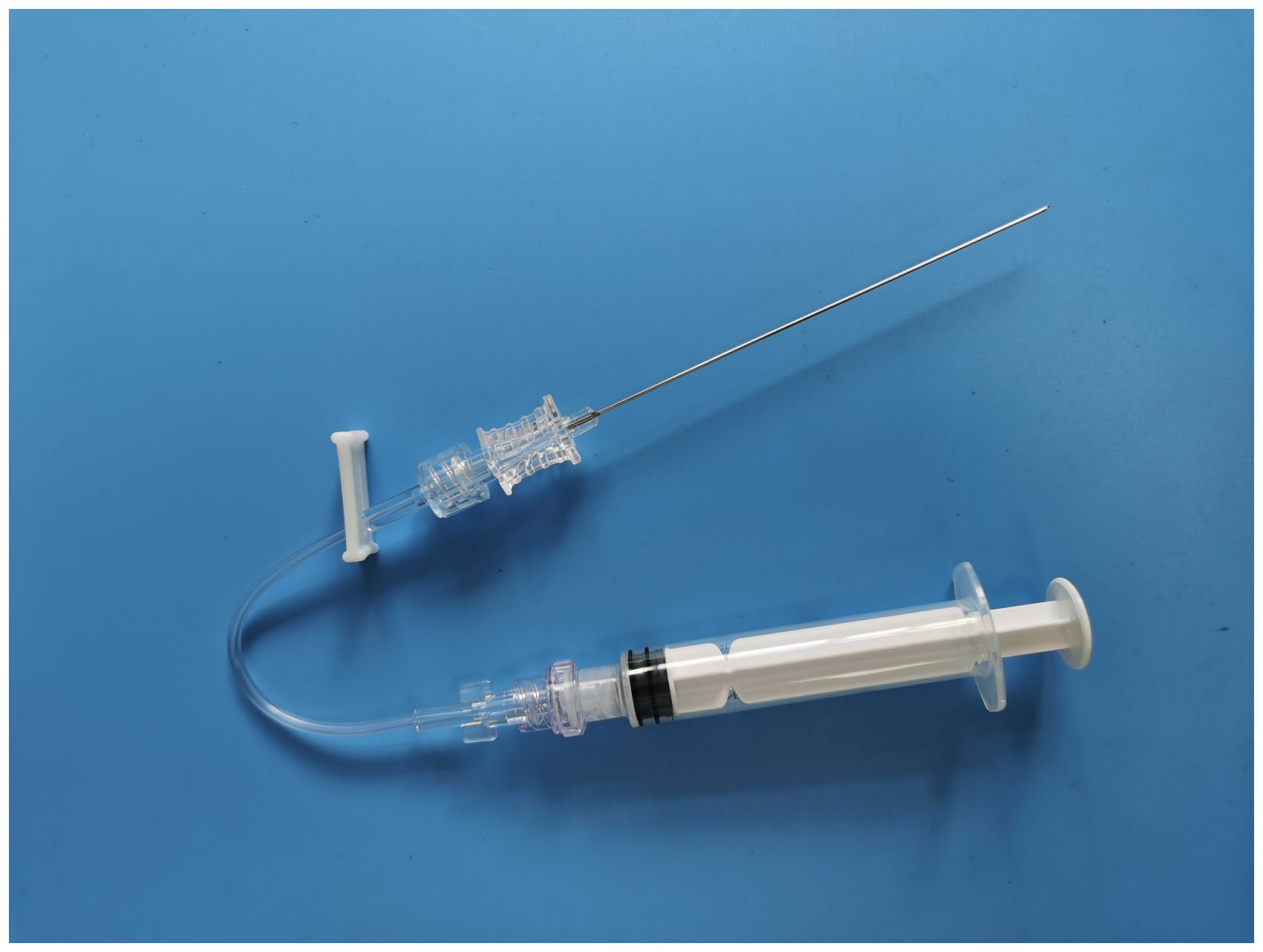
The three parts of Figure 1 connected.

The MIIM includes a septal needle-free closed infusion connector (designed with one-way flow control) with a screw mouth exactly matching the lumbar puncture needle at one end. This type of infusion connector is often used in routine vascular infusion. The infusion connector is connected between the lumbar puncture needle and the syringe (Figures 1, 2) when performing the MIIM. The detailed procedure of the MIIM is as follows:

1. After routine lumbar puncture, remove the stylet of the lumbar puncture needle, connect the injection connector through the screw port, and use a syringe to extract cerebrospinal fluid (CSF).
2. After confirming the flow of CSF, take another 10 mL syringe and inject 5 mL of air into the amperometer containing the solution of nusinersen (as stated in the instruction manual). Then, use the same syringe to withdraw 5 mL of the solution, while also extracting a small amount of gas accordingly.
3. As the connector will hold 0.34 mL of liquid, a small amount of gas will be injected into the connection between the lumbar puncture needle and the connector after the injection of nusinersen is completed to ensure that all the medication is injected into the subarachnoid space.
4. Connect a 10 mL syringe filled with nusinersen and a little air to the connector, and then push the medication at a relatively constant speed. During the process, pay attention to maintain the end of the syringe facing upwards to ensure that air is not injected into the subarachnoid space.
5. After the infusion of the medication is completed, inject a small amount of air that is already present in the syringe through the transparent connector pipeline. Pay attention to finish the infusion when the air reaches the connection between the lumbar puncture needle and the connector.

The applicability and safety of the MIIM were evaluated through clinical observation. Whether the entire amount of medication was successfully injected into the subarachnoid space of each patient, as well as possible adverse events such as fever, headache, low back pain, and other signs of nervous system infection after injection, were recorded accordingly.

We collected the opinions of the five experienced clinicians who were in charge of nusinersen injections regarding the advantages and disadvantages of the MIIM compared with the routine method, and the results are summarized in Table 1.

**Table 1:** Comparison of the MIIM with the routine method.

Differences between MIIM and routine method	Clinician No.				
	1	2	3	4	5
More accurate dose control				✓	✓
More constant injection rate	✓	✓	✓		
Better control of injection time	✓	✓			
Arms are more relaxed and comfortable during injection	✓	✓	✓		
Less risk of unstable puncture needle	✓	✓	✓		
More resistance when absorbing CSF through injection connector joined with puncture needle					✓

## Results

Since 2019, a total of 92 children with SMA have received over 500 injections of nusinersen using the MIIM at our hospital, including 28 cases of type 1, 45 cases of type 2, and 19 cases of type 3. Among them, five patients had fever after the injection, of which two developed symptoms of low-grade fever, mild headache, and back pain within 24 h after the injection, and all the symptoms remitted within 1 day and were evaluated to be related to nusinersen itself. One out of the five patients developed fever and cough 2 days after the injection and was subsequently diagnosed with upper respiratory tract infection (URI) and recovered soon after outpatient treatment of the URI. The remaining two patients developed fever and cough 2–3 days after the injection and were diagnosed with pneumonia and recovered soon after hospitalization. About 5% of the included patients developed low back pain, which lasted 2–3 days. No case developed any infection or experienced other adverse events related to the intrathecal injection, such as central nervous system (CNS) infections.

The five experienced clinicians reported the advantages and disadvantages of the MIIM (Table 1) compared with the routine method. The most reported advantages included successfully injecting the total dose of nusinersen with a more constant injection rate (3/5), less risk of an unstable puncture needle (3/5), and more relaxed arms of the operator (3/5), although one clinician felt resistance when absorbing CSF through the injection connector (1/5).

## Discussion

There have been reports on alternative methods focusing on reducing the difficulties in intrathecal injection for SMA patients with severe scoliotic spinal deformation and even those accepting spinal fusion surgery, including placement of intrathecal port catheter (6) or Ommaya reservoir (7), transforaminal intrathecal delivery (8), fluoroscopic lumbar puncture (9), and computed tomography-guided injection (10). To date, no MIIM especially for nusinersen treatment has been reported. The MIIM described in this study provides another novel injection choice besides the routine method, which may be helpful especially for some patients who need intrathecal injection under special circumstances.

The routine procedure of the MIIM is to insert the lumbar puncture needle into the subarachnoid space first, then pull out the stylet, and connect the needle to a syringe containing the medication to complete the injection procedure. However, this routine method has some disadvantages: first, the connection between the lumbar puncture needle and the syringe may become loose under some circumstances, resulting in drug leakage. For children, especially infants who have less control of their body, the part of the lumbar puncture needle inserted into the body may not be long enough to maintain the needle in the same position. Additionally, the injection speed is difficult to control.

The MIIM has several advantages for clinical practice. The septal needle-free closed infusion connector is connected to the lumbar puncture needle through a matched screw mouth, which creates a firm connection and avoids any possible drug leakage. Moreover, the septal needle-free closed infusion connector is designed with one-way flow control—injections follow the designated direction without outflow, further avoiding possible leakage. Also, with the connector, clinicians can effectively ensure the correct amount of medication by injecting a small amount of air to push the liquid to the connection of the lumbar puncture and the syringe. Additionally, the flexible pipe in the middle of the infusion connector reduces the pressure transmission, minimizing the chances of needle movement during the procedure. During the injection, the amount of tension on the operator’s arms could be reduced, which could provide more flexibility of the arms and reduce the psychological pressure on the operator. Finally, the flexible pipe of the infusion connector helps to better control the injection speed. Therefore, the MIIM makes the procedure of nusinersen administration easier and more precise in terms of the injection speed, control, and amount of injected drug. All the five performers of nusinersen injection preferred the MIIM to the routine method.

About 5% of the included patients developed low back pain that lasted 2–3 days, which could also be caused by routine lumbar puncture [the incidence rate was not higher than that for routine lumbar puncture (11)].

The MIIM is suitable for different types of SMA and is typically not influenced by the severity of SMA itself once the puncture needle is injected into the subarachnoid space. It is especially suitable for type 1 SMA patients whose low back muscles have reduced muscle tone, which may result in insufficient support for the inserted lumbar puncture needle, thereby increasing the possibility of the lumbar puncture needle entering the subarachnoid space to shift its position.

This MIIM is not limited to the administration of nusinersen, but is also applicable to drugs with strict injection doses and high injection speed requirements. The modified procedure is especially suitable for those patients who have less control of their body, especially pediatric patients.

This study had some limitations. First, it was a single center study, and multi-center studies should be conducted to further compare the applicability and safety of the MIIM with the routine method. Second, quantitative analysis needs to be performed in the subsequent studies.

## Data availability statement

The original contributions presented in the study are included in the article/supplementary material, further inquiries can be directed to the corresponding authors.

## Ethics statement

The studies involving humans were approved by Ethics committee of Guangzhou Women and Children’s Medical Center. The studies were conducted in accordance with the local legislation and institutional requirements. Written informed consent for participation in this study was provided by the participants' legal guardians/next of kin.

## Author contributions

YaZ and KZ carried out clinical treatment, designed the modified method, and drafted the manuscript. CL, MJ, ZZ, YuZ, MR, and SY prepared for materials. RZ and JC carried out clinical treatment. WC and LL supervised the research and revised the manuscript for intellectual content. All authors contributed to the article and approved the submitted version.

## Conflict of interest

The authors declare that the research was conducted in the absence of any commercial or financial relationships that could be construed as a potential conflict of interest.

## Publisher’s note

All claims expressed in this article are solely those of the authors and do not necessarily represent those of their affiliated organizations, or those of the publisher, the editors and the reviewers. Any product that may be evaluated in this article, or claim that may be made by its manufacturer, is not guaranteed or endorsed by the publisher.

## Abbreviations

MIIM: Modified intrathecal injection method
